# Comment on “Under-the-Flap Crosslinking and LASIK in Early Ectasia with Hyperopic Refractive Error”

**DOI:** 10.1155/2019/4986597

**Published:** 2019-07-01

**Authors:** Avi Wallerstein, Mathieu Gauvin, Mark Cohen

**Affiliations:** ^1^Department of Ophthalmology, Faculty of Medicine, McGill University, Montreal, QC, Canada; ^2^LASIK MD, Montreal, QC, Canada; ^3^Department of Surgery, Faculty of Medicine and Health Sciences, University of Sherbrooke, Sherbrooke, QC, Canada

We read with great interest the article by el-Khoury et al. “*Under-the-Flap Crosslinking and LASIK in Early Ectasia with Hyperopic Refractive Error*” [1]. It is encouraging to see clinical work with the goal of stabilizing the progression of early post-LASIK ectasia and improving visual outcomes. In their article, a combination of excimer laser ablation and corneal collagen crosslinking (CXL) was performed under an existing LASIK flap.

Sequential excimer laser ablation with CXL has been previously described for the management of cornea ectasia after LASIK [2–4]. The novelty of el-Khoury's article is applying this combination “under-flap” instead of performing a corneal surface treatment with epithelial removal.

While el-Khoury and colleagues included key references on CXL combined with excimer laser ablation in their article, we noted an important omission of the source reference that first introduced and described the “under-flap” approach to CXL in patients with early ectasia. The article “Under-flap stromal bed CXL for early post-LASIK ectasia: a novel treatment technique” was published in *Clinical Ophthalmology* in December 2016 [5]. This article is the first to describe and report on outcomes of performing CXL performed under a previous LASIK flap.

The unintentional omission of this reference might lead the readers to wrongfully believe that Dr. el-Khoury and colleagues were the first to describe an under-flap approach to CXL in the management of early post-LASIK ectasia. We would kindly ask Dr. el-Khoury to update his reference list so that the readers can be better informed of the origins of the *under-flap* method for CXL.

We would like to congratulate the authors for their contribution to the field.
